# Voices for change: inclusion of lived experience self-injury research, practice, education, and advocacy

**DOI:** 10.1080/00049530.2025.2456728

**Published:** 2025-02-02

**Authors:** Penelope Hasking, Amanda Aiyana, Sophie Haywood, Kassandra Hon, Katrina Hon, Sylvanna Mirichlis, Kirsty Stewart, Adrienne Wilmot, Stephen P. Lewis

**Affiliations:** a School of Population Health, Curtin University; b Curtin enAble Institute, Curtin University; c School of Allied Health, Curtin University; d Department of Psychology, University of Guelph

**Keywords:** Self-injury, NSSI, lived experience

## Abstract

**Objective:**

Despite gains in research knowledge, self-injury remains unduly and widely stigmatised. This can preclude people with lived experience from playing active and important roles in the field. In this paper, we discuss how people with lived experience can offer vital contributions in this regard.

**Method:**

Position paper based on narrative review.

**Results:**

According to the current and especially recent literature in the field, people with lived experience of self-injury can play significant roles as researchers, educators, clinicians, and advocates.

**Conclusion:**

Given the unique perspectives and strength people with lived experience of self-injury have to offer, their contributions to the field need to be harnessed and championed. This requires concerted efforts to address stigma and otherwise unhelpful discourses. In doing so, a more inclusive field with greater representation of people with lived experience can be realised. This, in turn, is conducive to advancing our understanding of self-injury and promoting the wellbeing of all people with such lived experience.

## KEY POINTS

**What is already known about this topic:**

1. Self-injury remains unduly and widely stigmatised.
2. People with lived experience are often reluctant to disclose this experience.
3. People with lived experience of self-injury can play significant roles as researchers, educators, clinicians, and advocates.

**What this topic adds:**

1. Grounded in a stigma resistance framework, we outline the opportunities and challenges experienced by individuals with lived experience working as researchers, educators, clinicians, and advocates.
2. We champion a more inclusive approach to embracing, rather than diminishing, the expertise they bring to their work.
3. We need to do better in educating grant reviewers, granting bodies, education providers, and clinical training programmes in the unique contribution people with lived experience can make to the field.

In this position paper, we discuss the roles that people with lived experience of non-suicidal self-injury (NSSI) can hold in academia, education, and clinical practice. In recent decades, NSSI has become a global public health concern. Defined as the deliberate, self-directed damage of body tissue without suicidal intent and for purposes not socially or culturally sanctioned (ISSS, 2018), self-injury is engaged in by approximately 8% of 8–12-year-olds, 18% of adolescents, 13% of young adults, and 5% of adults (>25 years; Barrocas et al., 2012; Farkas et al., 2023; Swannell et al., 2014). Self-injury is primarily used to cope with intense or unwanted emotions (Taylor et al., 2018), though is associated with a range of mental health concerns and represents a reliable, but weak, predictor of subsequent suicidal behaviour (Bentley et al., 2015; Kiekens et al., 2018; Ribeiro et al., 2016). Here we outline the different roles people can have as researchers, educators, clinicians, and advocates, discussing opportunities and challenges that come with these roles.

Recently, there has been a growing demand for inclusion of people with lived experience, including self-injury, in research and service provision. Funding bodies such as the National Health and Medical Research Council (NHMRC) engage consumers on their committees, in the peer review process for grants, and require inclusion of consumers in research projects (NHMRC, 2016). Guidelines have also been developed for the formation and support of mental health Lived Experience (Peer) workforces, to inform development, planning, delivery, and evaluation of mental health services (Byrne et al., 2021). There have also been calls for the further inclusion of people with lived experience of self-injury specifically, in research (Lewis & Hasking, 2019).

Beyond these “designated” lived experience roles, where an individual is actively sought for their lived experience views, there are many researchers, educators, clinicians, and advocates in “non-designated” lived experience roles. These individuals have the opportunity to bring their lived experience to these professional roles but can be silenced or experience stigma if their history of self-injury is known (Victor et al., 2022a). In this position paper, and framed through a stigma-resistance lens, we outline key opportunities and challenges experienced by individuals with lived experience across the following roles they may play: as researchers, educators, clinicians, and advocates. In doing so, we champion a more inclusive approach to embracing, rather than diminishing, the expertise they bring to their work.

## Self-injury stigma and stigma resistance

Self-injury is a highly stigmatised behaviour, with its volitional nature countering the biological imperative to not hurt ourselves often being a source of confusion for those unfamiliar with the behaviour (Staniland et al., 2021). The stigma associated with self-injury is present cross-contextually (e.g., general public, healthcare settings, educational settings) and has a profound impact on people who self-injure (e.g., shame, disclosure reluctance; see Staniland et al., 2021). Along these lines, people who self-injure have been described as “*weak*, *crazy*, *stupid”*, and are often met with directives to *“just stop doing it”* (Staniland et al., 2023), with no consideration for the function it serves or the difficulty inherent in spontaneously ceasing self-injury. Most self-injury research is deficit in its view of self-injury, focusing on individuals who self-injure lacking adequate or alternative coping strategies (Giordano et al., 2023), having poor emotion regulation skills (Christoforou et al., 2021), and an inability to tolerate distress (Slabbert et al., 2022). Increasingly though there has been advocacy for person-centred perspectives in pushing against self-injury stigma to highlight the strengths and value of people with lived experience of self-injury (Lewis & Hasking, 2021a; Victor et al., 2022c).

Stigma resistance is the capacity to resist or remain unaffected by stigma (Ritsher et al., 2003). Stigma resistance in the context of deflecting internalised stigma has been associated with quality of life, self-esteem, empowerment, hope, and recovery from severe mental illness (Dubreucq et al., 2022; Ritsher & Phelan, 2004). Stigma resistance at the peer and public level, can manifest as people with lived experience helping others fight stigma or educating the public to minimise public stigma (Firmin et al., 2017). For some, this includes disclosing one’s own lived experience in order to educate others. Despite recent calls for a more person-centred, strengths-based approach to understanding self-injury (Lewis & Hasking, 2021a, 2021b, 2023), its stigmatisation remains. It is perhaps for this reason that individuals who self-injure are reluctant to disclose this to others, both in personal and professional settings (Simone & Hamza, 2020). Taken together, the high degree of stigma, and reluctance to disclose self-injury, means that people who self-injure are not often considered to be present in, or capable of, professional roles in psychology.

## Lived experience researchers

Traditionally, the dominant paradigm of consumer involvement is to only involve people with lived experience of self-injury as research participants. The contribution of lived-experience research participants has been invaluable, providing critical expertise on topics that are germane to their experience and guiding directions for research (Lewis et al., 2019b; Wang et al., 2022); however, the value of lived experience perspectives from researchers themselves has long been overlooked. Despite ongoing encouragement for the inclusion of lived experience perspectives in research and service provision (e.g., World Health Organization, 2023), few researchers themselves have disclosed a history of self-injury, fearing stigma, being viewed as untrustworthy or selfish, and damage to their professional reputation (Devendorf et al., 2023). A recent survey of researchers in North America revealed that over 50% had conducted “self-relevant research” or what is termed, in a more derogatory sense, “me-search”. Of concern, psychologists and clinical trainees had more negative attitudes towards self-relevant research on mental health topics than physical health topics, and participants without lived experience made more stigmatising judgements of self-relevant research and researcher disclosure than people with lived experience (Devendorf et al., 2023).

The credibility of self-relevant research continues to be contested, both within academia and by the public (Altenmüller et al., 2021; Devendorf et al., 2023). Self-relevant research is often seen as subjective, leading to assumptions of researcher bias and generally poor science (Gardner et al., 2017). This stigma is particularly present in mental health research, perhaps due to the negative stereotyping of people with mental illness (Devendorf, 2022). Although these critiques generally reflect concern for the integrity of research and conceptions of what constitutes quality research, they rest on the assumption that research broadly is irrefutably objective. Beyond there being a lack of consensus in defining scientific objectivity (Van Dongen & Sikorski, 2021), objectivism like other paradigms exists within a socially constructed culture, and is rooted in European ideals (Niaz, 2018). We would argue that *all* research is subjective to a degree, not only through decisions made regarding choice of research questions, design, interpretation, and reporting, but by virtue of the researcher being interested in the topic.

As researchers, we play an active role throughout the research process regardless of whether the research is personally relevant (Van Dongen & Sikorski, 2021). Harnessing this active role could be instrumental not only in resisting stigma towards self-relevant research and self-injury broadly (Devendorf, 2022; Devendorf et al., 2023; Staniland et al., 2023), but also in contributing to quality research. Lived experience researchers are in a unique position to interpret and understand perspectives shared by their participants, and their connection to their research may facilitate intrinsic motivation in work that could be more purposefully prioritised (Devendorf, 2022; Lewis & Hasking, 2019).

There are, however, some important considerations in managing the impact of the active role of researchers, both for the integrity of the research and welfare of researchers. For example, the management of bias through reflexive practice is widely recommended (e.g., Jacobson & Mustafa, 2019), with professional peer support and mentoring suggested in the event of, and to prevent, potential distress from engaging in one’s research and/or from potential repercussions of self-disclosure in research. Research experiences may be emotional, distressing and/or triggering for researchers with lived experience (Dray et al., 2024; Lewis & Hasking, 2019) and researchers need to consider whether they are emotionally ready to engage in personally relevant research. In particular, there is a need for recognition that lived experience researchers should not have their experience exploited, should offer their experience when and how *they* see fit, and are provided with professional supervision to ensure that they are supported in their work both emotionally and professionally. This is particularly true for more junior researchers, who may have less experience balancing their roles as people who self-injure and research leaders.

We know that some researchers may disclose their lived experience status in some contexts (e.g., once their career is well-established) and not others (e.g., when they are a junior researcher) or with some colleagues (e.g., with their PhD students) but not others (e.g., other academic staff; Stirling & Chandler, 2021). Choosing when and where to disclose should always be a personal decision, though academic institutions should work to create environments in which researchers feel safe to disclose without fear of repercussions stemming from their disclosure. By way of creating transparent policies and procedures that are supportive of those with lived experience, universities can play an active role in stigma resistance. If guidelines are to be established, then these could emphasise that there should be no forward disclosures nor any negative consequences of disclosure for a researchers’ career at that institution. These collective steps are conducive to not only resisting stigma but also fostering a stigma-free working climate.

As suggested by Dray et al. (2024) the dual identity of lived experience researchers as both individuals who self-injure and professionals needs to be valued and respected. Lived experienced researchers in leadership positions have an opportunity to both influence the research agenda and provide role models to counter unhelpful discourses about who conducts research while also working to reduce stigma (Dray et al., 2024; Lewis & Hasking, 2019). Commensurate with this, a growing movement within broader academia is to formally recognise such roles. For example, the Canadian Institute of Health Research offers funding for projects specifically led by people with lived experience, a model we believe should be embraced in Australia. Some of the authors have been explicitly directed not to disclose their lived experience in grant applications for Australian competitive funding, based on the assumption that this would render the grant uncompetitive. This leaves many in the frustrating position of needing to demonstrate consumer and community involvement in the development of the grant, without explicitly disclosing that the research itself was informed by the lived experience of the primary investigator. These barriers can also foment self-stigma. Accordingly, a shift in the mindset of grant reviewers and granting bodies is required to resist stigma, thereby formally recognising the expertise and value a researcher with lived experience can offer.

## Lived experience educators

Many lecturers and tutors happen to have lived experience relevant to topics they teach, and some universities have created specialised roles for Lived Experience Educators. Lived Experience Educators have a designated role as outlined in the National Lived Experience (Peer) Workforces Workforce Development Guidelines (Byrne et al., 2021) – an initiative that developed out of the 5^th^ National Mental Health and Suicide Prevention Plan (National Mental Health Commission, 2021). The Framework emphasises that Lived Experience (Peer) workers draw on their personal or family/significant other/kinship experiences of distress, hope, and healing. These experiences combined with Lived Experience education, training, and peer supervision help people to develop their lived experience expertise. This lived experience expertise is the essential requirement to be a Lived Experience (Peer) worker.

If National and State guidelines of embedding Lived Experience Workers are followed, Lived Experience Educators can experience the positive benefits of sharing their expertise. A 2021 report published by CFE Research (Welford et al., 2021) states that as well as influencing systems change, there are positive benefits for the experts involved including developing confidence and feelings of self-worth and feeling pride in being able to contribute. Reframing negative past experiences as learning to drive positive change can be empowering and satisfying. Interaction between service users and workers humanises the individuals on both sides, challenging stigma and stereotypes and potentially reducing power imbalances (Welford et al., 2021).

The Valuing Lived Experience Program (VLEP) is an Australian-first initiative that has successfully involved Lived Experience Educators within a tertiary organisation. The VLEP was developed in 2015 by Lyn Mahboub Clinical/Professional Fellow: Lived Experience Academic, and Associate Professor Robyn Martin from Curtin University’s School of Allied Health. In a letter addressed to the Mental Health Commissioner on the 8 April 2022, The Mental Health Advisory Council, Chair, Margaret Doherty wrote:

It is the belief of MHAC that this program is so important and relevant that it should be core training for the mental health sector and in addition should be integrated into all practice delivery training. The aims of this program are to ‘meaningfully embed the voices of people with lived experience of mental distress, trauma, and use of health and community services into the education of students, academics, and professionals. (Doherty, 2022).

Programs such as the VLEP are vital for specialised education and training to gain the skills to safely share stories such that any negative effects involved in the emotional labour of composing and telling one’s story are minimised. Indeed, LeBlanc-Omstead and Kinsella (2023) note that telling one’s story was the most emotionally involved aspect of this work. For some, this involved “managing anxiety”, while others indicated this contributed to reliving difficult or “triggering” experiences.

Emotional labour is further compounded by perceived and enacted stigma. A 2016 Australian study of lived experience practitioners states that stigma and discrimination were reported as common experiences for all participants, with most accepting it as a natural or “‘normal’” part of working life. Consequently, some participants appeared to be desensitised or resigned to discriminatory treatment. Acceptance of stigma and discrimination was common:

…there’s always going to be people who are going to stigmatise … I guess you get conditioned to being treated that way.

Other participants expressed beliefs about lived experience educators needing to be thoughtful in the way they present themselves. Particularly, to be careful of not reinforcing what were perceived as low expectations from some professional colleagues. Similarly, several participants felt scrutinised by colleagues and had to overcompensate by always appearing professional or risk being seen as “‘unwell’”:

I do think I’ve had to overcompensate. I’m aware of how I dress, of how I move, of how I engage, that there is always the potential I will be misread as being inappropriate, and that being due to my lived experience rather than just a personality thing. (Byrne et al., 2016).

In addition to the emotional labour that can come with being a lived experience educator, there is risk of peer drift (MindAustralia, 2023). Peer drift occurs when peer practitioners do not feel comfortable in their peer role, and they begin to shift to a more medical treatment role. This is more common when a biomedical model is dominant, or in teams in which lived experience is not understood or valued. Similarly, epistemic injustice experienced by peer educators in the healthcare system can transfer to the teaching context because the expertise of people with lived experience is devalued in favour of “professional” knowledge (Okoroji et al., 2023). This devaluing of lived experience conflicts with the ethos of employing lived experience educators and should be actively addressed in any programme involving individuals with lived experience. Finally, lived-experience educators need to be aware of the “seduction of inclusion” or the pride and relief at being invited to the table, coupled with the temptation to be compliant, in order to avoid negative stereotypes associated with mental illness (Doherty et al., 2021). This compliance can lead people to conform with the “real” experts in the room, rather than openly sharing their experiences. Lived experience educators need to be comfortable and assured that they are the experts in their own experience and feel confident in sharing their voices.

Notwithstanding these challenges, the involvement of lived experience educators in psychology programmes offers much promise in offering relevant training to students. This is particularly true in psychology training in Australia, where students will not engage in clinical practice, and may not meet someone with lived experience in a professional capacity, for the first 4 years of their study. McAllister et al. (2023) recently reported that medical students would value the inclusion of stigma resistance in their university training, to better equip them to reduce interpersonal stigma and improve patient care. Working with an educator with lived experience offers an opportunity to resist stigma by way of bringing a human element into training, and to instal values of inclusion and respect among students. In a training project led by one of the authors, a health care professional noted:

Speaking and listening to someone with lived experience has allowed me to humanise people who self-injure.

Seeing people who self-injure as humans appears to be a fundamental attitude that should be integral to all psychology training programmes.

## Lived experience clinicians

The World Health Organization (2023) has championed for greater inclusion of lived experience voices in the context of service provision and mental health care. The inclusion of people with lived experience of self-injury ought to be part of this vision as they can play key roles as mental health professionals. Indeed, people with lived experience of self-injury are apt to have unique and often powerful insights into the nature of what it is like to live with self-injury on a daily basis, and thus have a sense of what can help clients to meet their individual needs and goals. This may be especially important when considering the difficulty involved with disclosing self-injury (e.g., Mirichlis et al., 2023; Rosenrot & Lewis, 2020), the ambivalence many feel about stopping self-injury (Gray et al., 2023), and its pervasive stigma (Burke et al., 2019; Staniland et al., 2021). Yet, the formal recognition of such lived experience in formal clinical roles is arguably easier said than done often due to stigma, which can impede people’s involvement in service provider roles in a variety of ways.

Taking on the role of mental health professional first requires people with lived experience to feel as though they can apply to and be accepted in relevant training programmes. Yet, prospective clinical psychology students – and likely those in other applied disciplines – may be actively discouraged from disclosing self-injury and other mental health difficulties in their applications (Devendorf, 2022; Victor et al., 2022a). This explicit silencing of lived experience may exacerbate self-stigma (Victor et al., 2022a).

Unfortunately, some of the concerns prospective applicants to applied psychology programmes have about disclosing parts of their experience are not unfounded. In the past, one-quarter of training directors of accredited American doctoral programmes have indicated that a past (*not current*) psychological impairment may preclude entry to the programme (Huprich & Rudd, 2004). More recently, there has also been evidence that people with lived experience of self-injury may encounter stigmatising and otherwise unhelpful attitudes and comments from people who hold power positions in graduate training programmes (e.g., Victor et al., 2022a; Victor et al., 2022b). While the extent to which such reports actively discourage people from pursuing careers of interest warrants empirical attention, it is reasonable to conclude that some individuals will be deterred from applying for postgraduate psychology programmes. In response to this, efforts have been made which offer solutions to foster more inclusion of people with lived experience of NSSI and other mental health challenges in psychology graduate programmes and allied fields (see Victor et al., 2022a; Victor et al., 2022b); drawing on these recommendations may prove fruitful for those involved in admissions and hiring processes.

The above notwithstanding, people with lived experience are indeed involved in training programmes and care provision after their training. Of note, more than 80% of respondents to a recent survey of applied psychology students, staff, and faculty reported mental health difficulties – including NSSI, with 48% reporting a mental illness diagnosis (Victor et al., 2022c). Although these numbers make it clear that people in applied psychology programmes – much like in *all* professions – experience mental health challenges, this does not mean that they feel accepted with regard to their lived experience, nor does it mean that they do not face barriers when applying to programmes, during their time enrolled, or when engaging in professional practice pursuant to graduation.

There is an apparent paradox among clinicians having their own experience of psychological distress; a clinician is expected to be non-judgemental, understanding, and empathic when it comes to a client’s experiences, but may be considered weak and unreliable if they have mental health concerns of their own (Victor et al., 2022c). In support of this are findings from a UK-based survey of 678 practicing clinical psychologists (Tay et al., 2018) which suggested that two-thirds of respondents had lived experience of mental illness, with many reluctant to disclose this at work due to concern about impact on their career and this eliciting shame. Similar reports have been made by individuals when contemplating whether they should, or if it was safe, to disclose self-injury in the context of their training or professional work (e.g., Lewis, 2016; Victor et al., 2022c).

Understandably, should a clinician experience mental health challenges, this has the potential to negatively impact their work and thus patient care – as would be the case for any person in any job. Findings from a recent study, however, suggest that although many trainees and professionals in applied psychology (e.g., counselling, clinical) report lived experience of mental health difficulties, they also indicate that this did not have an adverse impact on their work (Victor et al., 2022c). It may be that the potential for people’s work to be adversely affected is mitigated by some of the practices embedded in the training people receive. For example, in the context of service provision, clinicians can proactively navigate challenges through clinical supervision, consultation, and reflective practice. Such approaches represent some of the means by which any clinician biases or blind spots are intended to be addressed (Victor et al., 2022c). Clinicians may also seek support themselves, which can permit an opportunity to process and work through any impact their experience has on their work.

With that said, the presence of workplace stigma can thwart disclosure (McGrath et al., 2023) and one’s comfort in seeking support and resources. This may be especially so in relation to NSSI given the particular stigma it evokes. There are also likely to be unique contributors to stigma when it comes to self-injury (e.g., the presence of visible scarring, which may be seen by colleagues or clients). Taken together, there is potential for the creation of a feedback loop wherein clinicians with lived experience are unable to access appropriate mechanisms to manage wellbeing and issues impacting clinical care, worsening their wellbeing compared to their peers without lived experience. As the stigma tied to NSSI has been suggested to be greater than that attached to other mental health difficulties, as illustrated by recent research (Hamza et al., 2023), these potential consequences may be more pronounced when clinicians have a history of self-injury.

We would be remiss if we did not address the potential for clinicians and other colleagues in healthcare settings to stigmatise colleagues with lived experience. This raises some uncomfortable yet critical questions about people’s values, and whether those holding stigmatising views directed at colleagues are themselves able to provide an appropriately non-judgemental, empathetic space with clients in their own practice. Although beyond the scope of this paper, the stigmatisation of self-injury is pervasive and likely permeates some clinical settings; hence, addressing stigma will require conscious effort by the profession as a whole. Positive change to curb stigma will compel us to “walk the talk” of partnership with consumers and carers informed by genuine valuing of lived experience, including among our peers. In Jennifer White’s (2019) inspirational treatise on the transformative potential of an ethical, collaborative global suicide prevention movement, she challenges those involved to imagine other possible futures, grounded in a shared understanding of our interdependence and relational accountability. A shift in how we see ourselves, our colleagues with lived experience, consumers, and carers will help us collectively move towards a compassionate reimagining of how we understand and value our colleagues with lived experience of NSSI, suicidality, and mental illness.

Finally, when considering the role of clinicians with lived experience, it is important to consider one’s lived experience becoming known to healthcare consumers (who also have lived experience). To date, scant research is available regarding the consumer impact and perspective around receiving care from clinicians with lived experience of mental health adversity. One study suggests lay people generally prefer to seek out a clinician *without* lived experience of either their own personal therapy or suicidality – and will knowingly seek out treatment from someone with poorer clinical outcomes in order to avoid clinicians with their own lived experience (Rodriguez et al., 2023). In the context of self-injury, there is virtually no such research available. Nevertheless, there are several reports that people who have lived experience of self-injury prefer talking to and hearing from like-minded others (i.e., those who have “been there”) and may feel more comfortable sharing their experience with someone who “gets it”. For example, this is a common theme found in research examining how self-injury is discussed in online arenas (see Lewis & Seko, 2016; Lewis et al., 2019a; Pritchard & Lewis, 2023). How this translates to the clinical context remains unknown and may merit empirical attention.

When considering the lived experience of clinicians becoming known, questions also emerge regarding whether, when, and how a clinician should mention aspects of their own experience. Certainly, this would need to be carefully navigated and be done on one’s terms and at a time of readiness, much as it would for any form of disclosure from a clinician to a client. There is indeed a vast and broader literature on clinician disclosure that may be useful as a starting point in this regard (Zamir et al., 2022). For readers who wish to read more about disclosure by psychologists and other professionals with regard to self-injury, we encourage interested readers to consult some of the recent works in the field that address these matters in greater detail (e.g., Victor et al., 2022a; Victor et al., 2022b).

## Lived experience advocates

Advocacy has traditionally been used to modify laws, policies, practices, and influence health practices for individuals (Chapman, 2001). Lived experience advocates are in a unique position to be able to provide alternative perspectives to conventional medical services and provide a perspective of someone who has similar/shared experiences (Nelson et al., 2017). However, the acceptance of lived experience advocates in the self-injury space has been slow.

There are numerous online platforms devoted to advocacy for individuals who self-injure including an active community with a presence on social media platforms (e.g., Instagram, Tumblr, Reddit). The majority of these are focused on sharing positive experiences of recovery, building an understanding community, and offering stories of hope (Guccini & McKinley, 2022; Lewis & Seko, 2016; Lewis, et al., 2014). Young people, in particular, value online information as a source of help-seeking (Frost & Casey, 2016); this applies as well in the context of self-injury (e.g., Lewis et al., 2014). An analysis of narrative online blogs revealed that scars resulting from self-injury can play a critical role in stigma resistance (Kendall et al., 2021). Yet, information online can also pose potential risks. For example, some messages about self-injury are hopeless in nature and may reinforce the view that little can be done to stop it; in other instances, graphic descriptions or imagery can provoke upset or be triggering (for reviews, see Lewis & Seko, 2016, Lewis et al., 2019a; Pritchard & Lewis, 2023).

For this reason, social media giants such as Facebook/Meta and X (formerly Twitter) have implemented strict policies about what content can be posted (Perkins et al., 2021). While the intent of these policies is to protect viewers and avoid sensationalising or promoting self-injury, the policies are also there to explicitly prevent normalising the behaviour. TikTok’s policy regarding self-injury states: “To avoid normalizing, encouraging, or triggering self-harm behaviour, we do not allow imagery that depicts such behaviour, *regardless of the user’s intention of posting it* (emphasis added)”. One of the most effective ways to combat stigma concerns the parasocial contact hypothesis (Schiappa et al., 2005). Here, exposure to marginalised groups, including people with mental disorders, has been demonstrated to reduce stigma and prejudice towards these individuals (Corrigan et al., 2012). Recently, this has been applied to technology-based interventions (e.g., video games, virtual reality) to reduce stigma towards people with mental illness (Rodriguez-Rivas et al., 2023). The use of contact via digital technologies has also been highlighted as a potentially potent means to resist and address the stigma tied to self-injury (Lewis et al., 2021) and to provide advocacy for people with lived experience (Stirling & Lewis, 2024).

Policies such as TikTok’s ostensibly prevent this means of exposing people to individuals who self-injure and present a lost opportunity for education and advocacy. By blocking people from sharing their stories, it silences voices that already feel silenced which can foment shame and self-stigma (Hasking et al., 2023). Anecdotally we know individuals who have posted pictures of themselves gardening or playing with a pet dog, when they did not have their self-injury scars covered. Although the posts had nothing to do with self-injury these were removed by the site administrators for violating the self-injury policy. Such action not only poses a missed opportunity for normalisation and exposure but can leave individuals to feel even more stigmatised as a result of their self-injury. Moreover, such policies also do not align with extant evidence suggesting that online images of scars (as opposed to injuries) may not be triggering to people with lived experience of self-injury (Baker & Lewis, 2013).

Incorporating lived experience in self-injury discussions recognises that each person has a unique experience of self-injury and acknowledges people who self-injure as the experts in their own experiences (Hasking et al., 2023; Lewis & Hasking, 2023). Given the individualised experiences of self-injury, providing opportunities for people to share their experiences facilitates a deeper understanding of the functions, meanings, and outcomes of self-injury and may clarify any misconceptions that people may hold about self-injury (Lewis & Hasking, 2023). Indeed, people more familiar with self-injury (e.g., being in contact with someone who has engaged in self-injury) are more likely to adopt inclusive and accepting attitudes towards self-injury (Burke et al., 2019). Similarly, advocates who share their lived experiences may feel empowered to find meaning in their self-injury and become more accepting of their self-injury experiences through the process of self-reflection; indeed, there are examples of such reports from people who have shared such experiences publicly (Lewis, 2016). Such empowerment may help people view their self-injury as a symbol of resilience and strength, and reduce any internalised shame associated with their self-injury history (Hasking et al., 2023; Lewis, 2016). Indeed, people begin to develop more hope and positive views of themselves as they navigate their personal recovery journey, which strengthens their capacity to work towards their life goals and cope with obstacles along the way (Hasking et al., 2023). Thus, advocating for lived experience in understanding self-injury presents a desirable approach to promote self-injury recovery via self-acceptance and empowerment.

Although incorporating lived experience perspectives into advocacy efforts can be invaluable, there are some key considerations to bear in mind. In their dialogue paper, Stirling and Chandler (2021) explore their relationship with the label of “self-injury advocate”, reflecting a seeming assumption that people with lived experience should be advocates regardless of their own intentions. This assumption presents a paradox, in that it can actively impede a lived experienced individual’s agency (i.e., in the choice whether to engage in advocacy), by expecting them to engage in an activity to promote agency. Though assuming an advocate role can have potential benefits, it can leave individuals privy to strains such as those on their time and emotional wellbeing, as discussed in Lombardo’s (n.d.) online blog about her experience as a lived-experience mental health advocate.

Such emotional labour and burnout are widely cited among carers and professionals supporting those with mental health difficulties (Mercuri et al., 2021). Understanding and communicating one’s boundaries as an advocate is therefore of great importance, aligning with considerations raised by Test et al. (2005) in their framework for self-advocacy. Test et al. (2005) highlight knowledge of oneself, including strengths, needs, and goals, as well as knowledge of their rights and resources as being central to self-advocacy. The considerations by Test et al. (2005) encourage individual agency of advocates, though this alone is not sufficient to ensure agency, in part due to power imbalances potentially faced by advocates, stigmatisation of self-injury, the potential personal and professional repercussions of disclosing one’s lived experience (Devendorf, 2022; Staniland et al., 2023), and the diversity of experiences that ought to be represented.

## Conclusion

Meaningfully engaging people with lived experience requires the ongoing commitment to shift from tokenistic to more genuine modes of engagement, and to ensure diversity in skills and experience. Throughout this manuscript, we have discussed the roles of researchers, educators, clinicians, and advocates as if they are mutually distinct professions, though it should be noted that these experiences are often intersectional, and all of these are to be valued. Where then lies the distinction between professional and personal? Boundaries are of course important, but at times there is a sentiment akin to Carol Hanisch’s “the personal is political” (1969/2006). That is, at times we must bring our personal lives to light in order to make overdue, needed, and lasting change. There are very valid critiques of allowing one’s personal life to infiltrate one’s working life, and vice versa. However, the opportunity to separate them is often a matter of privilege; not everyone has the choice to go to work without seeing experiences similar to that of their personal life reflected. Managing these different roles is important for psychology professionals with lived experience of self-injury and should form a core part of psychology training.

Throughout this manuscript, we have highlighted the role that stigma plays in silencing professionals with lived experience. How then do we challenge this stigma to allow people to use their experiences in their work (if they choose to do so)? We need to do better in educating grant reviewers, granting bodies, education providers, and clinical training programs in the unique contributions people with lived experience can make to the field. Encouraging more people with lived experience to serve in leadership roles may go some way to resisting the systematic stigma we observe but will take brave leaders to disclose their own experiences. Researchers and clinicians with lived experience of self-injury are slowly coming to the fore (Devendorf et al., 2023; Lewis, 2016) and we support and encourage them in their endeavours. There is also work to be done in understanding the views of psychology professionals who have experience of self-injury. Of note, we must explore gender differences in response to disclosure. Self-injury is often, incorrectly, assumed to be a “female” behaviour. As a result, men who self-injure have been dismissed or considered to simply be “letting off steam”. Men may thus face even more stigma when drawing on their lived experience in a professional context (Lewis, 2016).

In conclusion, we recognise that individuals with lived experience of self-injury are already successfully working as researchers (and research leaders), educators, clinicians, and advocates. Considering these individuals to be weak, unreliable, or incapable simply flies in the face of the evidence. As a profession we need to embrace the experiences of our colleagues and provide a space for their expertise to be recognised, valued, and required. In this way we can foster meaningful stigma resistance and move the field forward to be more inclusive, and perhaps more relevant, when working to understand and respond to people with lived experience of self-injury.

## Positionality statement

All authors have lived experience of self-injury, mental illness, and/or caring for someone with a mental illness. Our daily battle of balancing our lived experience with our professional roles as researchers, educators, clinicians, and/or advocates has informed our opinions presented in this article and interpretation of the literature. We recognise the need for substantial change within the field of applied psychology with respect to how lived experience is recognised and included. We believe that acknowledging lived experience of mental health difficulties within our professional communities is an integral component of efforts to improve diversity, equity, and inclusion within psychological science.
